# No evidence of association between Atherosclerosis, risk factors for cardiovascular disease and human T-cell lymphotropic virus type 1(HTLV-1) infection

**DOI:** 10.1186/1742-4690-12-S1-P30

**Published:** 2015-08-28

**Authors:** Glauco Moniz de Aragão Dória, Viviana Olavarria Gallazzi, Ney Boa-Sorte, Maria Fernanda Rios Grassi, Bernardo Galvão-Castro

**Affiliations:** 1 Centro Integrativo e Interdisciplinar de HTLV (CHTLV), Escola Bahiana de Medicina e Saúde Pública, Salvador, Bahia, Brasil; 2 Laboratório Avançado de Saúde Pública, Centro de Pesquisas Gonçalo Moniz, Fundação Oswaldo Cruz/Bahia, Brazil

## 

HTLV-1 induces a persistent infection leading to an increased production of inflammatory cytokines. HIV, another retrovirus that causes systemic inflammation, is associated with atherosclerosis. However, few is known about HTLV-1-infection and atherosclerosis. To determine the prevalence and risk factors associated with atherosclerosis in patients infected with HTLV-1. a cross-sectional study involving 54 HTLV-1-infected patients (24 asymptomatic and 30 HAM/TSP), was carried out at the CHTLV between 2012 to 2013. Sociodemographic and cardiovascular risk factors were evaluated (age, sex, arterial hypertension, hypercholesterolemia, diabetes mellitus and smoking). Patients were submitted to Doppler echocardiography of both carotid and vertebral arteries to measure intimal-media layer. The association between intima-media thickness (IMT) and HAM/TSP diagnosis, HTLV-1 proviral load, low-density lipoprotrein cholesterol (LDL-c) or ultrasensitive C-reactive protein was determined. The mean age of patients was 57.1±12.3 years, 72.2% were women. Clinical atherosclerosis (IMT≥1.5mm) was found in nine patients (16.7%) and subclinical atherosclerosis (IMT>1 and <1.5) in 10 patients (18.5%). Atherosclerosis was more frequent in women (41%) compared with man (20%). Median age was significantly higher in patients with atherosclerosis (p=0,2). The mean of LDL-c level was 140.11±43.25 mg/dl (ranging from 49 to 287mg/dl). No significant differences were observed between the mean LDL-c level in individuals with subclinical (126.10±38.95mg/dl) and with atherosclerosis diagnosis (144.±43.87mg/dl), compared with individuals with normal IMT (143.21±44.65mg/dl), (p=0.4175). There was no correlation between HTLV-1 proviral load and IMT. No association was found between HTLV-1 infection and clinical or subclinical atherosclerosis as well as with the risk factors for cardiovascular diseases. A positive association between atherosclerosis and age was observed.

